# The Love Canal Story Is Not Finished

**DOI:** 10.1289/ehp.12310

**Published:** 2009-02

**Authors:** Richard Clapp

**Affiliations:** Department of Environmental Health, Boston University School of Public Health, Boston, Massachusetts, E-mail: rclapp@bu.edu

In this issue, Gensburg et al. (2009) summarize the mortality experience of > 6,100 former residents of Love Canal, New York, over the period 1979–1996. Love Canal became a household word 30 years ago when outraged residents, led by Lois Gibbs and the Love Canal Homeowners Association, demanded attention to the apparent adverse effects of hazardous waste exposures on their children’s health (Boston University School of Public Health 2004). Love Canal was evacuated between 1978 and 1980, and property owners were compensated in the first such widely publicized creation of environmental refugees in the United States. Several books and documentaries have described the process and the responses of the various parties involved, including the New York State Department of Health (Boston University School of Public Health 2004; Levine 1982).

The Love Canal saga was called a “warning signal” for other communities that could be experiencing the same types of exposures and similar effects on children’s health. As a result of this evacuation and other similar instances in contaminated communities around the United States, the Senate Environment and Public Works Committee and other legislative committees held hearings that led to the passage of the Superfund legislation in 1980. The trust fund created by this legislation paid for cleanup of the most dangerous contamination sites, and its amendments and reauthorization in 1986 created the Agency for Toxic Substances and Disease Registry (ATSDR; Atlanta, GA) to conduct health studies of residents in exposed communities, among other things. The New York State Department of Health used funds from the ATSDR to pay, in part, for the study by Gensburg et al. (2009).

Early studies by researchers at the Roswell Park Memorial Institute (Buffalo, NY) suggested an increased number of stillbirths, birth defects, and other adverse reproductive outcomes in Love Canal children (Goldman et al. 1985). Initial evaluation of cancer incidence suggested a possible increase in respiratory cancer, but it was left to later investigators to examine this more thoroughly. Over the years since the initial controversy about health impacts, community representatives have expressed concern that the scientific information has been part of a “politically inspired cover-up” (Levine 1983). The mortality study by Gensburg et al. (2009) is part of the Love Canal Follow-up Health Study, an attempt to use existing records to understand the health consequences of living near Love Canal between 1940 and 1978, with community involvement and the advice of a prestigious expert advisory committee. Additional results are available in the Project Report to the ATSDR (New York State Department of Health 2008) and will be the subject of future published articles.

The results of the mortality study are limited by several factors, which Gensburg et al. (2009) describe in the “Discussion” of their article. The most obvious limitation, which is common to most retrospective studies of community exposures, is the inability to assess exposure before 1978 and reliance on qualitative estimates. The authors note that “exposure misclassification may have occurred, obscuring possible associations.” Another common limitation is the reliance on death certificate information, with its attendant incompleteness and inaccuracy with respect to certain causes of death. The two most striking findings—increased deaths from acute myocardial infarction, and external causes, such as suicide and motor vehicle accidents—are less susceptible to inaccurate reporting than, for example, specific cancers.

The relatively short follow-up period and relatively young average age of the participants through 1996 led Gensburg et al. to conclude that further follow-up “could reveal patterns that are not yet apparent.” The full story about the health impacts of living near Love Canal is yet to come. Given the importance of this community in the history of environmental health over the past three decades, it is well worth the effort required to understand and honestly report the full story.

## Figures and Tables

**Figure:**
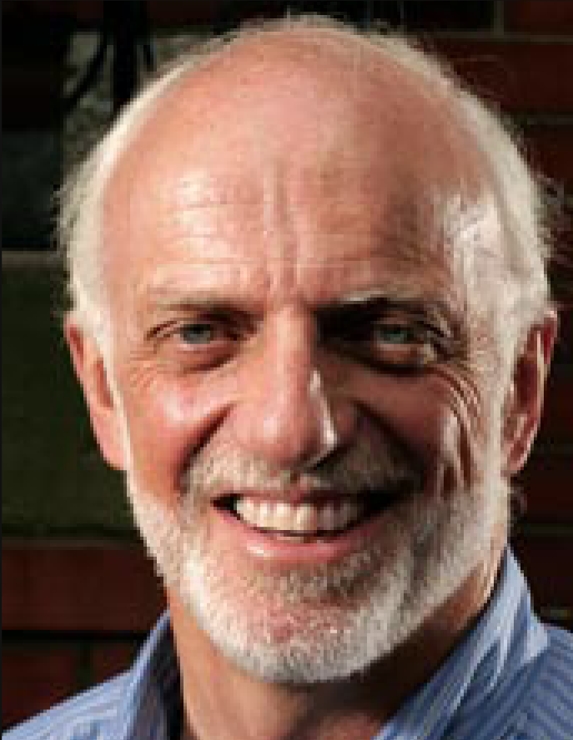
Richard Clapp
